# Determination of the quantized topological magneto-electric effect in topological insulators from Rayleigh scattering

**DOI:** 10.1038/srep07948

**Published:** 2015-01-22

**Authors:** Lixin Ge, Tianrong Zhan, Dezhuan Han, Xiaohan Liu, Jian Zi

**Affiliations:** 1 Department of Physics, Key laboratory of Micro and Nano Photonic Structures (MOE), and Key Laboratory of Surface Physics, Fudan University, Shanghai 200433, China; 2 Department of Applied Physics, Chongqing University, Chongqing 400044, China; 3 Collaborative Innovation Center of Advanced Microstructures, Nanjing 210093, China

## Abstract

Topological insulators (TIs) exhibit many exotic properties. In particular, a topological magneto-electric (TME) effect, quantized in units of the fine structure constant, exists in TIs. Here, we theoretically study the scattering properties of electromagnetic waves by TI circular cylinders particularly in the Rayleigh scattering limit. Compared with ordinary dielectric cylinders, the scattering by TI cylinders shows many unusual features due to the TME effect. Two proposals are suggested to determine the TME effect of TIs simply by measuring the electric-field components of scattered waves in the far field at one or two scattering angles. Our results could also offer a way to measure the fine structure constant.

The scattering of electromagnetic (EM) waves by small particles is a common optical phenomenon^1^,^2^. According to the size of scatterers, it can be classified into Rayleigh (for scatterer sizes much smaller than the wavelength) and Mie scattering (for scatterer sizes comparable to the wavelength). The most known example of Rayleigh scattering is the blue color of the sky wherein the scattering intensity by the gas molecules in the atmosphere varies inversely with the fourth power of the wavelength. The scattering of EM waves depends not only on the scatterer size and geometry but also on the EM properties of scatterers. In addition to conventional dielectric ones, scatterers made of emerging artificial materials have received broad interest in recent years and many extraordinary scattering properties have been revealed^3^,^4^,^5^,^6^,^7^,^8^,^9^,^10^. For instance, the scattering of an object composed of metamaterial in the far field could be reduced to zero (cloaking)^3^,^4^, or even transformed to other objects of different appearance^5^. For a subwavelength nanorod consisting of multiple concentric layers of dielectric and plasmonic materials, its scattering cross-section can far exceed the single-channel limit, leading to superscattering^6^. For particles with magnetic responses, many unusual EM scattering properties such as zero forward scattering has been proposed and confirmed experimentally^7^,^8^. Even being one-atom thick, arrays of graphene nano-disks could nearly completely absorb infrared light at certain resonant wavelengths^9^,^10^.

As an emerging phase in condensed matter physics, topological insulators (TIs) have been of great interest in recent years^11^,^12^,^13^ due to their exotic properties. In the bulk, TIs resemble ordinary insulators possessing a bulk energy gap. However, their surface states are gapless (metallic) and protected topologically by time-reversal symmetry. TI materials have been theoretically predicted and experimentally confirmed in several material systems^14^,^15^,^16^,^17^,^18^. In additional to exotic electronic and transport properties, TIs show many unusual EM properties^19^,^20^,^21^,^22^,^23^. For instance, a point charge atop the surface of a TI can induce an image magnetic monopole^19^. In a TI thin film, there exist a giant magneto-optical Kerr effect and an interesting Faraday effect with a universal rotation angle defined by the fine structure constant^20^. From the Kerr and Faraday angles in a TI thick film, one can even determine the half-quantized Hall conductance of the two TI surfaces independently without knowing the material details^21^. On TI surfaces, the surface plasmon modes can even couple to spin waves, forming interesting hybridized spin-plasmon modes^22^. In two TI plates, Casimir forces could even be switched to be repulsive^23^ although they are usually attractive in two ordinary dielectric plates. All these interesting phenomena stem from the topological magneto-electric (TME) effect arising from the unusual EM response in TIs^24^.

Here, we theoretically study the scattering of EM waves by circular TI cylinders, particularly in the Rayleigh scattering limit. Unusual scattering properties due to the TME effect are revealed. In three-dimensional TIs, the EM response can be described by a Lagrangian consisting of a conventional Maxwell term and an additional term related to the TME effect^24^, 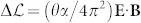, where **E** and **B** are the electric and magnetic fields, respectively; 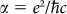 is the fine structure constant; and *θ* = (2*p*+ 1)*π* with *p* being an integer is a quantized angular variable to characterize the TME effect, known as the axion angle in particle physics^25^. By breaking the time-reversal symmetry at the surface (e.g., coating a TI with a thin magnetic layer), a surface energy gap will open up so that the value of *θ* can be specified definitely^24^,^26^. Indeed, *θ* gives a half-quantized Hall conductance *σ_xy_* = (*p* + 1/2)*e*^2^/*h* which can be viewed as the origin of the TME effect. In TIs, the propagation of EM waves can still be described by conventional Maxwell's equations. However, owing to the presence of the TME effect the constitutive relations in TIs should be modified as 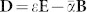 and 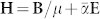
24, where **D** and **H** are, respectively, the electric displacement and the magnetic field strength; *ε* and *μ* are, respectively, the dielectric constant and magnetic permeability; and 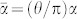 is a quantized quantity in units of the fine structure constant. Note that the effective description of the modified constitutive relations of TIs applies only for the photon energy 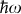 much smaller than both the bulk and surface energy gaps, where *ω* is the angular frequency of EM waves. Compared to conventional media such as anisotropic ones^27^,^28^,^29^, the scattering of EM waves by a TI cylinder differs in an additional contribution resulting from the TME effect, basically a surface and topological effect that gives rise to many unique and novel quantum phenomena^19^,^20^,^21^,^22^,^23^. For instance, the EM responses of an extremely thin film made of the conventional medium can be neglected for non-resonant cases. However, the EM scattering caused by the TME effect can still exist even the thickness is very small since it's a surface effect intrinsically^30^.

From the modified constitutive relations, which are a manifestation of the TME effect, two observations can be made. Firstly, the fine structure constant enters and therefore this fundamental physical constant characterizing the strength of EM interaction might be determined solely by optical measurements^20^,^21^. Secondly, the optical measurement could also be utilized to identify the TIs^31^,^32^. Other properties derived from this TME effect have also been investigated^33^,^34^. In the present work, our central purpose is to determine the quantized TME effect.

## Results

### Scattering of EM waves by TI cylinders

**Figure 1:**
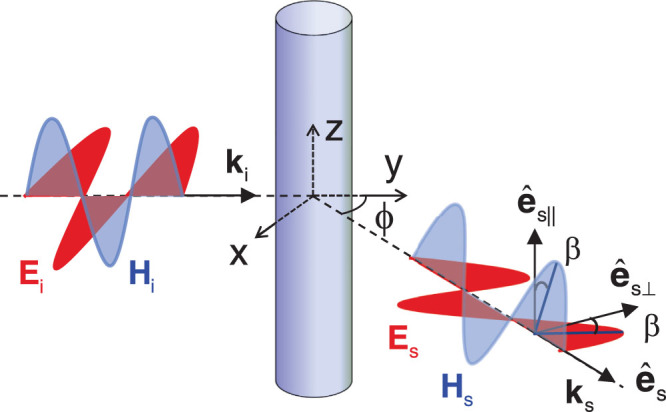
Schematic view of a circular TI cylinder placed along the *z* axis. The subscripts *i* and *s* denote incident and scattered waves, respectively. TE waves with a wave vector 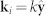 are incident perpendicularly to the TI cylinder, where 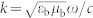. Another coordinate system is introduced to describe scattered waves in the far field with the orthonormal basis vectors: 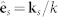 (**k***_s_* is the wave vector along the wave normal of scattered waves and lies in the *x*-*y* plane); 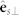 lies in the *x*-*y* plane but perpendicular to both 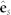 and 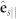; and 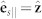. Note that **k***_s_* makes a scattering angle *ϕ* with the *y* axis. Both the electric and magnetic fields of scattered waves lie in the 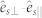 plane but may rotate by the same angle *β* due to the TME effect.

The system under study is schematically shown in Fig. 1. A circular TI cylinder with a radius *r* is placed along the *z* axis. We focus on transverse electric (TE) waves (with the magnetic field along the TI cylinder), which are incident perpendicularly to the TI cylinder in this study. Transverse magnetic (TM) incident waves (with the electric field along the TI cylinder) can be discussed similarly. The dielectric constant and magnetic permeability of the TI are denoted by *ε* and *μ*, respectively; and those of the background are *ε*_b_ and *μ*_b_. The axion angle of the TI is *θ* = (2*p* + 1)*π* while the background takes a trivial axion angle *θ* = 0 for simplicity. To break the time-reversal symmetry on the surface, the TI cylinder is coated with an ultrathin magnetic layer which plays almost no direct role in the EM wave scattering since its thickness is much smaller than both the radius of the TI cylinder and the wavelength of EM waves considered^35^. The EM scattering by this magnetic layer will be further discussed.

Based on the standard multipole expansion theory^1^,^2^, we can solve the scattering problem of EM waves by a circular TI cylinder with the modified constitutive relations and the conventional boundary conditions at the boundary between the TI cylinder and the background. The scattering coefficients {*a_n_*} and {*b_n_*}, related respectively to the electric and magnetic multipoles of order *n*, can be obtained (see Appendix for details). With these scattering coefficients, scattering properties of the TI cylinder can be obtained accordingly. It can be verified that *a*_−*n*_ = *a_n_* and *b*_−*n*_ = *b_n_*, similar to those in ordinary dielectric cylinders^1^,^2^. It should be mentioned that {*a_n_*} and {*b_n_*} are polarization-dependent. In other words, there exist two independent sets of the scattering coefficients, {*a_n_*_,TE_, *b_n_*_,TE_} and {*a_n_*_,TM_, *b_n_*_,TM_}. For an incident wave with an arbitrary polarization, its scattering properties can be discussed since it can be decomposed as a linear combination of TE and TM waves.

**Figure 2:**
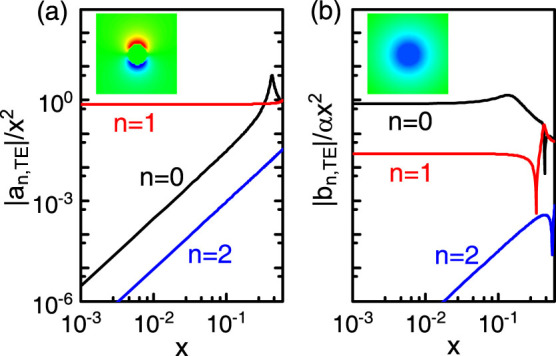
Scattering coefficients of TE incident waves for a TI cylinder in air (*ε*_b_ = *μ*_b_ = 1) with *ε* = 30 and *μ* = 1. The axion angle *θ* = *π* is taken for the TI. Note that in (a) |*a_n_*_,TE_| is normalized by *x*^2^ and in (b) |*b_n_*_,TE_| is normalized by *αx*^2^. In the Rayleigh limit 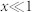, only the terms |*a*_1,TE_|, |*b*_0,TE_| and |*b*_1,TE_| are in the order of *x*^2^. The radial and *z* components of the electric fields that correspond to *a_n_* and *b_n_* are shown in the insets of (a) and (b).

Compared with ordinary dielectric cylinders, extra contributions resulting from the TME effect appear in both {*a_n_*} and {*b_n_*}, leading to many unusual scattering properties. For example, for an ordinary dielectric cylinder a TE incident wave cannot excite the magnetic multipoles because *b_n_*_,TE_ = 0 (not valid for *b_n_*_,TM_ generally). However, for a TI cylinder, *b_n_*_,TE_ does not vanish in general, implying that the magnetic multipoles can be excited. The underlying physics lies in the TME effect, whereby an electric (magnetic) field can induce a magnetic (electric) polarization. In Fig. 2, the scattering coefficients of a TI cylinder for TE incident waves as a function of the size parameter *x* = *kr* is shown. In general, the electric multipoles give much larger contributions to the scattering than the magnetic multipoles. In the Rayleigh scattering limit (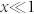 and 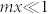 with 
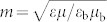
), for TE incident waves it can be shown that only the following scattering coefficients have the order of *x*^2^,
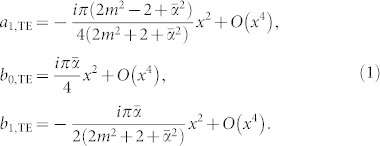
All other scattering coefficients have the order of *x*^4^ or higher, and can be hence neglected in Rayleigh scattering. In other words, in Rayleigh scattering only the electric dipole, as well as the magnetic monopole and dipole play roles in the scattering of TE incident waves. The corresponding electric fields are shown in the insets of Fig. 2. In the Mie scattering regime (*x* ~ 1), however, both the electric and magnetic multipoles will contribute to the scattering. We note that at certain frequencies resonant peaks, known as Mie resonances^1^,^2^ corresponding to the resonant excitations of the electric or magnetic multipoles, appear. For example, the peak at *x* = 0.425 in |*a*_0,TE_| corresponds to the resonant excitation of the electric monopole while the peak at *x* = 0.135 in |*b*_0,TE_| implies the resonant excitation of the magnetic monopole. In addition to resonant peaks, there exist sharp dips in |*b_n_*_,TE_| showing an *anti-resonance* behavior. The underlying physics is that at the dips a TE incident wave cannot induce a transverse magnetic field, leading to *b_n_*_,TE_ = 0, i.e., the absence of the TME effect at the dips. For TM incident waves on the other side, it can be shown that only the scattering coefficients *a*_0,TM_, *a*_1,TM_, *b*_0,TM_, and *b*_1,TM_ have the *O*(*x*^2^) term, and all other coefficients are in the order of *x*^4^ or higher. In fact, it can be verified that *a*_0,TM_ = *b*_0,TE_, *a*_1,TM_ = *b*_1,TE_, 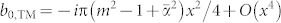, and 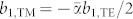. Different from TE incident waves, a TM incident wave can excite the electric monopole in Rayleigh scattering.

### Properties of the amplitude scattering matrix

To obtain the fields of scattered waves in the far field, an amplitude scattering matrix *T* is usually introduced which relates the electric field of scattered waves to that of incident waves^1^,^2^
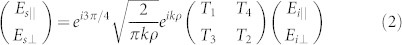
where *E*_||_ and *E*_⊥_ are the components of the electric field parallel and perpendicular to the TI cylinder, respectively; and *ρ* is the radial distance from the center of the TI cylinder in the *x*-*y* plane. From the definitions in Fig. 1, 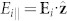, 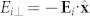, 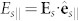, and 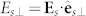. The elements of the amplitude scattering matrix *T* are given by 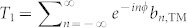, 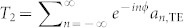, 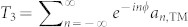, and 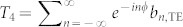. Obviously, the elements *T*_2_ and *T*_3_ are associated with the electric multipoles while *T*_1_ and *T*_4_ are related to the magnetic multipoles. In ordinary dielectric cylinders, the condition *b_n_*_,TE_ = 0 for TE incident waves leads the scattering matrix *T* to be diagonal; i.e., *T*_3_ = *T*_4_ = 0. In TI cylinders, however, *T* is not diagonal in general since *b_n_*_,TE_ may not be zero owing to the TME effect.

**Figure 3:**
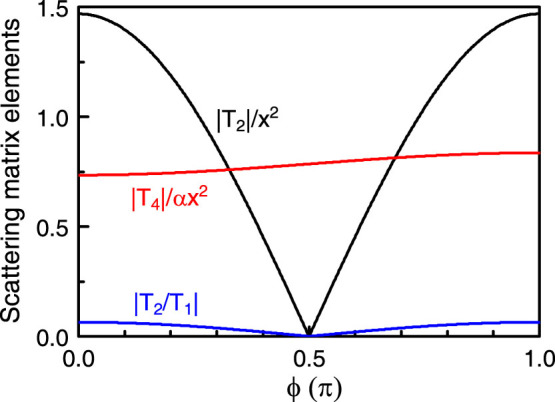
Scattering matrix elements in the Rayleigh scattering limit as a function of the scattering angle *ϕ* for a TI cylinder in air with *ε* = 30 and *μ* = 1. The axion angle is taken to be *θ* = *π*. Note that |*T*_2_| is normalized by *x*^2^ and |*T*_4_| is normalized by *αx*^2^.

In the Rayleigh scattering limit, it can be shown that the scattering matrix elements *T_i_* can be simplified to the following forms
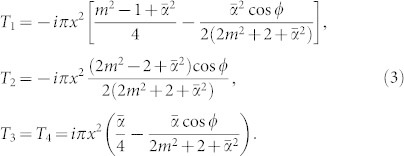
Clearly, the scattering matrix elements *T_i_* depends not only on the scattering angle *ϕ* but also on the axion angle. In Fig. 3, the scattering matrix elements as a function of *ϕ* are shown. The element |*T*_2_| depends strongly on *ϕ* showing a linear relation with cos *ϕ* in Rayleigh scattering. As a result, |*T*_2_| has two maxima at *ϕ* = 0 and *π*, corresponding to the forward and backward scattering, respectively. It vanishes at *ϕ* = *π*/2. By analyzing the origin, the element has a dominant contribution from a_1,TE_, namely, the electric dipole. In contrast, the element |*T*_4_|, which is linearly proportional to 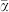 in Rayleigh scattering, shows a rather weak dependence on *ϕ* since there are two contributions from *b*_0,TE_ and *b*_1,TE_, associated with the magnetic monopole and dipole, respectively.

From the amplitude scattering matrix, information on scattering properties can be inferred. For instance, for a TE incident wave the scattered waves in general are still dominantly TE-polarized for the scattering angle away from *ϕ* = *π*/2, although there is a small rotation of the polarization due to the TME effect. This is because |*T*_4_| is much smaller than |*T*_2_| except for *ϕ* in the vicinity of *π*/2. Around *ϕ* = *π*/2, the polarization of the scattered waves undergoes a drastic change, and the scattered wave becomes purely TM-polarized at *ϕ* = *π*/2. This is a strong manifestation of the TME effect. For a TM incident wave, however, the scattered wave is always dominantly TM-polarized even at *ϕ* = *π*/2. This is why in this study we focus on TE rather than TM incident waves. From the scattering matrix, the measure of the TME effect is associated with the ratio |*T*_4_/*T*_2_| (|*T*_3_/*T*_1_|) for TE (TM) incident waves. Note that |*T*_1_| is much larger than both |*T*_2_| and |*T*_3_| for any *ϕ*. Thus, the ratio |*T*_3_/*T*_1_|, basically a manifestation of the TME effect, is more than an order of magnitude smaller than |*T*_4_/*T*_2_| which is related to TE incident waves.

### Determination of the quantized TME effect

To explore a quantitative determination of the TME effect, we can conduct Rayleigh scattering experiments with TE incident waves as schematically shown in Fig. 1. In the far field, both electric-field components of scattered waves |*E_s_*_||_| and |*E_s_*_⊥_| are measurable quantities and we can thus define a measurable quantity *R*(*ϕ*) = |*E_s_*_||_/*E_s_*_⊥_|. For TE incident waves in the Rayleigh scattering limit, 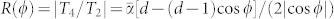, where 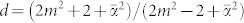 is weakly dependent on 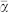 since 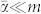 and is basically a bulk parameter. By neglecting the 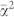 terms in *d*, we can obtain
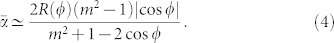
This offers a simple way to determine 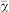 if *R*(*ϕ*) is measured at a certain scattering angle *ϕ* and the material parameter *m* is known. Note that 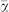 cannot be determined at *ϕ* = *π*/2 from Eq. (4) since *T*_2_ = 0. Thus, a scattering angle *ϕ* ≠ *π*/2 should be chosen if using Eq. (4). From Eq. (2) and *T*_4_, however, 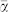 can still be determined at *ϕ* = *π*/2 as
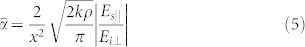
provided that |*E_s_*_||_/*E_i_*_⊥_| is measured, and the radius of the TI cylinder, the wavelength of the TE incident waves, and the distance between the TI cylinder and the detector are known.

Obviously, the one-angle measurement of 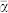 based on either Eq. (4) or (5) is dependent on material parameters such as *m* or *x*. To achieve a determination that is independent of material parameters, we can do the measurement twice at two different scattering angles, *ϕ*_1_ and *ϕ*_2_. With the two observables *R*(*ϕ*_1_) and *R*(*ϕ*_2_) and eliminating the material-dependent quantity *d*, 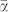 can be expressed as
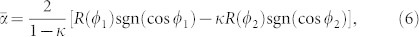
where *κ* = cos *ϕ*_2_(1 − cos *ϕ*_1_)/[cos *ϕ*_1_(1 − cos *ϕ*_2_)] is a parameter depending only on the scattering angles. The to-be-measured 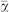 is now only a function of *ϕ*_1,2_ and *R*(*ϕ*_1,2_). As in the one-angle measurement based on Eq. (4), the scattering angle of *π*/2 should be avoided.

Equations (4)–(6) are the most important results in this study. Although the one-angle measurement based on Eq. (4) or (5) is simple, it is, however, dependent on material parameters since we have to know the material parameter *m* at the frequency of the incident waves or the radius of the TI cylinder. In contrast, the two-angle measurement based on Eq. (6) is material-independent. It needs only the measured quantity *R*(*ϕ*) at two scattering angles.

### Finite-size effects

**Figure 4:**
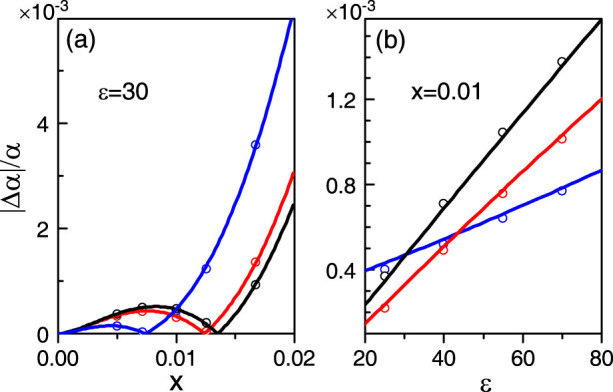
Deviation function with Mie effects fully taken into account. We choose *θ* = *π*, and three different sets of scattering angles *ϕ*_1,2_ = 90° + *δϕ*_1,2_. The black, red and blue curves correspond to (*δϕ*_1_, *δϕ*_2_) = (−12°, 7°), (−15°, 5°) and (−9°, 9°), respectively. (a) |Δ*α*|/*α* as a function of the size parameter *x* with *ε* = 30 fixed. (b) |Δ*α*|/*α* as a function of the bulk dielectric constant *ε* with *x* = 0.01 fixed. The circles show the corresponding numerical results (COMSOL Multi-physics).

To estimate the accuracy of the fine structure constant *α* determined in this optical measurement, we take the axion angle *θ* = *π* with no loss of generality and introduce a deviation function
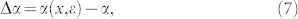
where *α*(*x*, *ε*) is no longer a constant but a function defined by Eq. (6), in which the quantity *R*(*ϕ*) = |*E_s_*_||_/*E_s_*_⊥_| is now calculated by the rigorous Mie theory without taking the Rayleigh limit. In Fig. 4(a), two scattering angles are chosen to be *ϕ*_1,2_ = 90° + *δϕ*_1,2_; and the deviation, |Δ*α*/*α*|, as a function of the size parameter *x* with *ε* = 30 fixed, is shown. For the three sets of scattering angles shown in Fig. 4(a), |Δ*α*/*α*| is in the order of 10^−4^ even for *x* ~ 0.01. We should note that the deviation function is weakly dependent on the choice of the scattering angles as the size effects taken into account. Fig. 4(b) shows |Δ*α*/*α*| as a function of the dielectric constant with *x* = 0.01 kept. The deviation function increases linearly with the bulk dielectric constant of TIs. However, it can still be in the order of 10^−4^ as the scattering angles are chosen appropriately even for *ε* = 80. These results calculated by Mie theory are further confirmed by the numerical simulations using a commercial software (COMSOL Multi-physics), as shown by the coloured circles in Fig. 4 correspondingly.

## Discussions

From 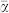 determined by Eqs. (4)–(6), the axion angle *θ* could be directly inferred, from which the half-quantized Hall conductance of the surface of the TI cylinder can be obtained. From the obtained 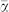, it also offers a way to measure the fine structure constant *α* since the axion angle is quantized. Practically, such Rayleigh scattering experiments can be conducted in the microwave regime. The radius of TI cylinders should be of the order of micrometers or tens of micrometers which well satisfies the Rayleigh-scattering-limit condition 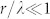, and the condition that the penetration depth of the electronic states 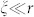. We note that *ξ* can be about a few nanometers for certain TIs as the topologically non-trivial gap is large^36^,^37^. Furthermore, for a typical value of the surface gap *E_g_* ~ 10 meV^24^, the incident photon energy in the microwave regime is much smaller than *E_g_*. To reduce the influence of incident waves, scattering angles around *π*/2 are suggested.

Another point should be mentioned is that the doping level^20^,^38^ in TIs we considered is in the gap. As the surface states are gapped, the axion angle is quantized and the “axion electrodynamics” can be applied to describe the EM responses of the system. To produce a surface gap, we need to introduce an insulating magnetic layer coating on the TI surface^35^,^39^. The role that the magnetic layer plays^20^,^24^ in the EM scattering should be rigorously restricted in the total EM scattering. Since the magnetic layer can be as thin as several nanometers^35^, the optical distance of this layer is extremely small in the microwave regime. Note that the magnetic layer itself may generically induce a Faraday rotation in addition to the surface electronic states in TIs. However, for the ultrathin ferromagnetic layer, its magneto-optical response can be insignificant and further compensated by an additional ferromagnetic film with opposite magnetizations^20^. Another relevant possibility is to introduce an anti-ferromagnetic layer^39^ surrounding the TI.

In summary, we studied Rayleigh scattering of EM waves by circular TI cylinders by using a multipole expansion theory. Based on the unconventional scattering features, two proposals were suggested to measure the quantized TME effect. The two-angle measurement has a promising feature of material-independence. Our proposal offers a way to determine the axion angle or a method to measure the fine structure constant.

## Methods

### Multipole expansion

Considering a time-harmonic EM wave with frequency *ω* incident perpendicularly to an infinite circular cylinder, this scattering problem can be solved analytically by the standard multipole expansion theory^1^,^2^. The incident, scattering and internal EM field can be expanded by the vector cylindrical harmonics
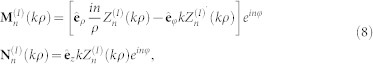
where *n* is an integer, *ρ* is the radial distance and *φ* is the azimuth angle, *k* is the corresponding wavevector, and 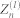 represent the Bessel function of the first kind and the Hankel function of the first kind for *I* = 1 and *I* = 3, respectively.

The system under study is shown in Fig. 1. We should note that the scattering angle was defined previously as *ϕ* = *π*/2 − *φ*. The wavevector in the background is 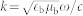. By using the vector cylindrical harmonics, the incident and scattered electric fields, and the internal electric field inside the TI cylinder can be expanded in terms of the vector cylindrical harmonics:
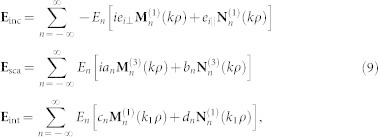
where *a_n_* and *b_n_* are the scattering coefficients, 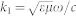 is the wavevector inside the TI cylinder, and *E_n_* = *E*_0_/*k* with *E*_0_ being the electric-field amplitude of the incident wave. *e_i_*_⊥_ and *e_i_*_||_ are the polarization-vector components of the incident wave with *e_i_*_⊥_ = 1, *e_i_*_||_ = 0 standing for the TE polarization (electric field perpendicular to the cylinder), and *e_i_*_⊥_ = 0, *e_i_*_||_ = 1 for the TM polarization (electric field parallel to the cylinder).

The boundary conditions at *ρ* = *r* read
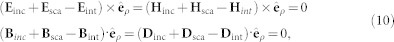
and the constitutive relations for TIs are given by 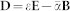, 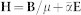
24. Here, 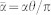, where *α* is the fine structure constant and *θ* = (2*p* + 1)*π* is the axion angle with *p* being an integer. The scattering coefficients can be found to be the following form:
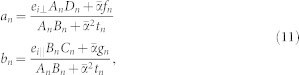
where *A_n_*, *B_n_*, *C_n_*, and *D_n_* are the same as that for the ordinary dielectric cylinders^1^, given by
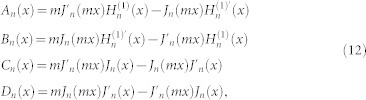
where *x* = *kr* is the size parameter and 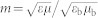. The auxiliary functions *f_n_*, *g_n_*, and *t_n_* are defined as follows:
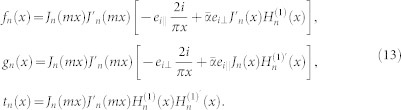
The scattering coefficients *a_n_* and *b_n_* are associated with the electric and magnetic multipoles of order *n*, respectively. It can be verified that *a_n_* = *a*_−*n*_ and *b_n_* = *b*_−*n*_, similar to those for the ordinary dielectric cylinders^1^,^2^. For the ordinary dielectric cylinders (*θ* = 0 or 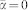), the scattering coefficients *a_n_* and *b_n_* are reduced to the conventional ones^1^,^2^ as expected. Note that for the incident waves there are two independent polarizations, TE and TM waves; as a result, there exist two sets of scattering coefficients, namely, {*a_n_*_,TE_, *b_n_*_,TE_} and {*a_n_*_,TM_, *b_n_*_,TM_}.

### Rayleigh limit

In the Rayleigh limit, i.e., 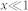 and 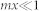, the scattering coefficients can be expanded by the Taylor series. If only the terms up to the order of *x*^2^ are kept, *a_n_* and *b_n_* for the TE-polarized incident waves are shown in Eq. (1). Obviously, in the Rayleigh limit only the electric dipole, as well as the magnetic monopole and dipole contribute to the scattering for the TE incident waves, while the electric monopole cannot be excited. For the ordinary dielectric cylinders, however, only the electric dipole dominates in the Rayleigh limit.

For TM-polarized incident waves, the scattering coefficients in the Rayleigh limit are also shown in the previous section. And we found that, in addition to the electric dipole, magnetic monopole and dipole, the electric monopole can be excited for TM-polarized incident waves compared to the TE-polarized incident waves.

## Author Contributions

The idea of this research was conceived by D.Z.H. and J.Z.; L.X.G. and X.H.L. performed analytical derivations and numerical calculations; T.R.Z. assisted in the analyzing and discussion of the results; D.Z.H. and J.Z. prepared the manuscript; All authors commented on the manuscript.
